# The International Medical Congress and the Veterinary Profession

**Published:** 1887-04

**Authors:** 


### THE INTERNATIONAL MEDICAL CONGRESS AND THE VETERINARY PROFESSION.

Are duly qualified members of the Veterinary Profession admitted to membership of the International Congress? is the question which, in September last, Dr. Ward, F. R. C. V. S., of Baltimore, proposed to the General Secretary of that body. An affirmative answer appears so naturally to be expected that the majority of thinking men who take an interest in those sciences which promote the welfare of humanity would consider absurd such a proposition. We are inclined to the same opinion, but the experience of the above-named eminent veterinarian would seem to justify the question.

In reply, the secretary stated that the question, being a new one, required reference to the Executive Committee, as no single officer could decide it.

What action was taken by the committee is not known, as Dr. Ward has not yet received, as ordinary courtesy would demand, any notification of the decision arrived at.

We presume that the hesitancy on the part of the committee is due to the fear of allowing entrance to men unfit to become members of an association of such high standing, and not to any reluctance to accept a union with the veterinary profession. No human physician can, to-day, deny the advantages to be derived from co-operating with the veterinarian in the field of scientific research.

*Apropos* of this subject are the following remarks made by Prof. Woodhead, M. D., at a meeting of the National Veterinary Association in Edinburgh, in reply to a vote of thanks accorded him for a paper he had read: "He could say from his own point of view, that it had been a very great pleasure to him and a very great profit; he had derived a very great deal of knowledge which he could not have otherwise derived, from engaging with Prof. McFadyean, V. S., in this kind of work." "One felt," he said, "how close the two professions were—how closely associated were medical science and veterinary science; and he felt that if they would only meet oftener and interchange ideas more freely, it would be of great advantage to both sciences."

On the continent, there is a tendency in this direction visible in many of the medical societies.

That a change of feeling in this country will soon occur, there is not the slightest doubt, and we predict that the near future will behold the two sciences marching hand in hand in the path of progress, and then it will be seen what good work has been accomplished by the science of comparative medicine and surgery. C.

---

Tuberculosis in the Grouse.—Dr. Morton Grinnell gives a history of some cases of the above disease in a late number of *Forest and Stream*.

Commenting on the importance of the discovery of the tubercle bacilli by Koch, of the dangers to which we are exposed of taking these germs into the body by means of our food and drink,cow's milk for instance, the Doctor refers to an article in the October number of the journal by Dr. J. B. Sutton on Avian Tuberculosis. The conclusion arrived at by the latter is that tuberculosis in birds is not developed by infection from man, as Nocard thinks, but is due to the formation and multiplication of this tubercule bacillus in congenial soil. The cases reported by Dr. Grinnell in which the grouse died after a confinement of six or eight weeks, and in which *post-mortem* examination revealed the existence of true tubercle bacilli, and the resulting lesions corroborate this view, although he does not think that the transmissibility from the sputum of tuberculous patients is a settled question.

There is no doubt, however, of the necessity of keeping the aviaries, barnyard, etc., well cleaned and disinfected. C.

---

Veterinary Society Meetings.—Philadelphia has, during the last month, gone through an era in her veterinary history, which we are sure will be of benefit both to the profession of Pennsylvania and to that of the whole country. The details of the meetings of the Pennsylvania State Veterinary Medical Association, on March 14th, and of the United States Veterinary Medical Association, on March 15th, will be found in the Society proceedings elsewhere. Among the best discussions, that on the danger of feeding tuberculous meat and milk, must assuredly bring some good result.

These meetings were held in the amphitheatre of the veterinary department of the University of Pennsylvania. The first was one of the largest since the organization of the association, two years ago; it was marked by better attendance, and the proceedings assumed rather more of a professional character than has been permissible at the earlier meetings, when the adoption of constitution and regulations, and the difficulty of defining the qualifications for membership occupied much of the time. The United States meeting can be regarded as a great compliment to Philadelphia. There was gathered a more complete and representative assembly of veterinarians than have before been together in this country. They came from Massachusetts in the East, and from beyond the Mississippi in the West; from Canada in the North came its most distinguished veterinarian to greet his Republican neighbors.

At a dinner held by the association, in the Clover Club room of the Hotel Bellevue, before parting, the visiting members were met and welcomed by their local colleagues, by the President of the Philadelphia County Medical Society, by several of the editors of the daily newspapers, and by other prominent citizens. President Liautard was most happy in conducting the social proceedings and toasts from the chair, while Prof. McEachran, Prof. Salmon and Prof. Lyford, on the part of the profession, and Mr. M. P. Handy, Dr. Cohen, Mr. Megargee and Dr. Osler, on the part of the citizens, vied with each other, among the many others, in bright responses, which showed that the necessary feeling ot good-fellowship, and recognition by the public of the veterinary profession, is rapidly developing.

The germs of veterinary medicine have had good soil on the American Continent, but have been sparsely strewn and frequently have been much dried before sowing. Let us hope that the crops will soon be good.

H.

---

Notes on the Diseases of Birds' Beaks.—Since publishing my paper in The Journal "On the injuries of the beak in birds and the method of repair," I have received a very interesting specimen, further illustrating this subject, from Mr. William Dutcher, of New York City, Supt. for the District of Long Island, for the Committee on Bird Migration of the American Ornithologists' Union. The specimen in question is that of a common blue-bird (*sialia sialis*, ♂.), collected by Mr. Dutcher, on the 2d of November, 1886, at Locust Valley, Queens Co., N. Y., it having been shot out of a flock of eight others.

This bird was suffering from a peculiar disease of the mandibles, it being the first example of anything of the kind that has ever fallen under my observation. Both bills are profoundly involved, but only as far back as the normal limiting line of feathers, which do not seem to be in the least way affected by the proximity of the diseased condition. It seems to have consisted in a low, chronic inflammation of the external superficies of the osseous mandibles, under the influence of which they were slowly crumbling away, as was also the horny theca that covered them. Indeed, the superior mandible is already two millimeters shorter than the lower one, and both show in their roughened appearance the inroads made by the disease; the nasal apertures are here filled in, apparently by the dried discharges.

The cause of such a disease as this is unknown to me, and possibly it is entirely confined to the class aves; I would add that the specimen is in excellent plumage, and its feet are perfect, and present no evidence of disease of any kind whatever. As I did not see the body of the bird, I am unable to say how well it was nourished.

R. W. Shufeldt, M. D.

*Fort Wingate, N. Mex.*

